# Bilateral Renal Hilar Nutcracker Phenomenon in a Male Cadaver

**DOI:** 10.15388/Amed.2024.31.1.6

**Published:** 2024-02-27

**Authors:** Dibakar Borthakur, Rajesh Kumar, Vidhu Dhawan, Rima Dada

**Affiliations:** 1 All India Institute of Medical Sciences, New Delhi, India; 2 All India Institute of Medical Sciences, Patna, India

**Keywords:** Renal hilar nutcracker phenomenon, renal hilum, renal artery, renal vein, inkstų hilarinis spragtuko fenomenas, inkstų sienelės, inkstų arterija, inkstų vena

## Abstract

Nutcracker phenomenon (NCP) typically refers to the entrapment of the left renal vein (LRV) between the aorta and the superior mesenteric artery. Similar to the classic NCP, the renal vein can also get entrapped between the segmental branches of the renal artery at the renal hilum, which has been referred to as ‘renal hilar nutcracker phenomenon (RHNP).’ During routine dissection of a male cadaver of 67 years, the renal veins of both sides at the renal hilum were seen between the segmental branches of renal arteries, which we identified as the ‘renal hilar nutcracker phenomenon.’ The disposition of the rest of the perihilar structures was normal. ‘Renal hilar nutcracker phenomenon’ can have similar clinical presentation like that of the nutcracker phenomenon. Hence, knowledge of such anatomical variation at the renal hilum is desirable.

## Abstract (it)

Terminu „spragtuko fenomenas“ įprastai apibūdinamas kairiosios inkstų venos įstrigimas tarp aortos ir viršutinės mezenterinės arterijos. Panašiai į klasikinį „spragtuko fenomeno“ atvejį, inkstų vena taip pat gali įstrigti ir tarp inkstų arterijos segmentinių atšakų ties inkstų sienelėmis. Šis reiškinys vadinamas inkstų hilariniu spragtuko fenomenu ir angliškai trumpinamas RHNP. Per 67 metų vyro lavono skrodimą buvo pastebėta, kad inkstų venos abiejose pusėse ties inkstų sienelėmis buvo išsidėsčiusios tarp inkstų arterijų segmentinių atšakų. Mes tai įvardijome „inkstų hilariniu spragtuko fenomenu“. Visos likusios perihilarinės struktūros išsidėstymas buvo normalus. Inkstų hilarinis spragtuko fenomenas kliniškai gali pasireikšti panašiai į spragtuko fenomeną. Todėl būtų naudinga įgyti daugiau žinių apie šią anatominę inkstų sienelių variaciją.

## Introduction

Blood vessels can get entrapped easily between adjacent vessels or other structures within the close packed abdominal cavity. Such a condition is the nutcracker phenomenon (NCP) which refers to the entrapment of the left renal vein (LRV) [1]. Typically the entrapment of LRV occurs between the abdominal aorta and the superior mesenteric artery (SMA) which is the classic NCP or anterior NCP. LRV can get entrapped between the aorta and the bodies of lumbar vertebrae, which is described as posterior NCP [2]. There are still many other reported varieties of LRV entrapment due to extrinsic compression from adjacent structures. The nutcracker syndrome (NCS) is a term specially reserved for NCP with frank clinical symptoms [1]. The clinical presentation of NCS occurs due to LRV entrapment with consequent changes in the hemodynamics of the LRV which secondarily causes outflow obstruction in the LRV. Similar to the entrapment of LRV in NCP, either of the RVs can get entrapped at the RH, when an anomalous segmental branch of the RA surrounds or encircles the RV. Such a condition was first identified by Sawant D A et al., 2015 and they described the condition as ‘renal hilar nutcracker phenomenon’ (RHNP). Though this phenomenon is very rare, bilateral RHNP was found in a cadaveric case and described here, wherein the RVs were lying between the segmental renal arteries at the RH.

## Case Report

**Figure 1:**
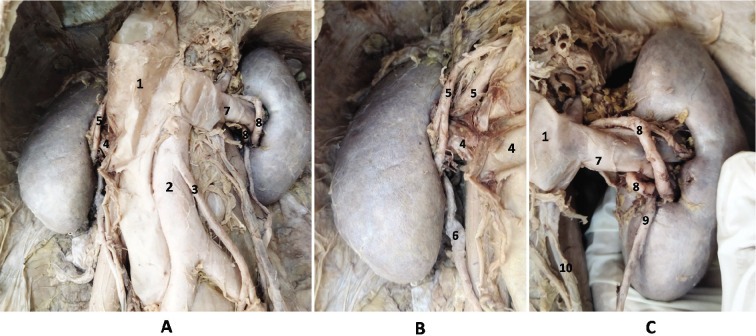
(A–C). Dissected kidneys and related retroperitoneal structures on the posterior abdominal wall showing the bilateral RHNP; 1 – inferior vena cava, 2 – abdominal aorta, 3 – inferior mesenteric artery, 4 – right renal vein, 5 – right renal artery branch, 6 – pelvis of right kidney, 7 – left renal vein, 8 – left renal artery branch, 9 – pelvis of left kidney, 10 – left gonadal vein draining into left renal vein

During routine dissection of a male cadaver of 67 years at the All India Institute of Medical Sciences, New Delhi, we observed an unusual relationship of renal vessels at the RH of both the kidneys. Institutional guidelines for the use of human cadaver in teaching and research were followed. Pararenal fat was removed in the paravertebral gutters and the anterior layer of the renal fascia was exposed. The renal fascia was split open by a vertical incision and perirenal fat around the kidneys and the suprarenal glands were removed carefully. Further meticulous dissection was carried out to expose the renal perihilar structures [3]. RVs on both the sides were seen to be sandwiched between the anterior and the posterior segmental branches of the RAs (Figure 1A). The right RA originated from the abdominal aorta approximately at the transpyloric plane, divided into two segmental branches 4 cm away from the right RH, which then ran posterior to the inferior vena cava (IVC) to reach the right RH. The anterior segmental branch on the right side divided further into two smaller branches both of which then arched anterior to the right RV and entered into the right RH in a plane anterior to the entry of the posterior segmental branch of right RA (Figure 1B). The left RA originated from the abdominal aorta at the transpyloric plane, ran laterally posterior to the left RV and divided into two segmental branches 3.5 cm away from the left RH. The anterior segmental branch on the left side bifurcated into two branches both of which spiralled the left RV anteriorly and entered the left RH lying in a plane anterior to the entry of the posterior segmental branch of the left RA (Figure 1C). On both the sides the RVs at the RH were in a position where they can get sandwiched between the segmental branches of the RAs. No other gross anomaly in the structures of the urinary system and perirenal vasculature was noted. As per the documented health record of the cadaver obtained during body donation, all the parameters related to renal profiles were found to be within normal limits and cause of death in the cadaver was not related to any renal disease.

## Discussion

The earliest records about the NCP were seen in the anatomical descriptions of *Grant*, 1937 [4]. El-Sadr and Mina during the 1950s described the NCP. *Chait et al*. and *Schepper et al*. in 1971–1972 coined the term NCS (5). The common clinical presentations of the NCS are left flank pain or abdominal pain, hematuria, varicocele in male and symptoms of pelvic congestion in females, etc. Management can be conservative or surgical such as laparoscopy and endovascular interventions [6, 7]. Several atypical varieties of NCS have been reported in the literature, where compression of the LRV occurred from vessels or structures other than the aorta and SMA [8]. Similarly right sided NCS secondary to a gravid uterus has also been reported [9]. With respect to anatomical and surgical aspects, the typical arrangements of the structures at the RH are RV, RA and the renal pelvis arranged from before backwards. The RA usually gives off five segmental branches just before entering the RH. Anatomical variations of the branching pattern of the segmental RAs as well as the variations of the renal hilar structures are not infrequent. The disposition of the two segmental branches of the RAs and the RVs as described in present case report can be considered as a nutcracker point, where the two segmental arteries represent the two limbs of the nutcracker. Thus it appears as if the RVs are in a vulnerable position for compression by the two segmental branches of the RAs. This type of unusual arrangement of structures at the renal hilum was first described by Sawant D A et al., 2015 and they termed the entity as the RHNP [10]. A recent study by Nautiyal A et al., 2023 identified three cases of RHNP [4]. The salient findings of these two studies are tabulated in Table 1. All these previous findings were found in cadaver. Clinical cases with this type of finding have not been described in literature as per our knowledge. It is quite possible that RHNP might cause subsequent entrapment of RV at the RH, and can present with clinical symptoms similar to the presentation observed in NCS. Considering no distinct nomenclature coined till date for this vascular pattern at the RH, we agree with the observations of Sawant D A et al., 2015 and reiterate that this particular vascular arrangement at the RH can be well labelled as RHNP.

**Table 1:** Erstwhile studies describing the renal hilar nutcracker phenomenon and their notable observations.

Authors	Study type	Population and sample size	Observation
*Nautiyal A et al. 2023* (4)	Autopsy and radiological	Indian, 49 cases	In 3 cases, the left RV was arched closely in front by looping anterior inferior segmental branch of left RA
*Sawant D A et al., 2015* (10)	Autopsy	American, single case	Entrapment of LRV observed between left anterior inferior and posterior branches of left RA.

Since this type of findings has not been well described in clinical cases, the condition might go unnoticed even after detailed work up in a patient. So the surgeon or nephrologists dealing with renal pathology should have a keen eye to diagnose any abnormality in renal hilum. The narrowing of the renal vein (RV) and consequent renal hilar dilatation in NCS is readily demonstrable in routine diagnostic tests such as the Doppler ultrasonography, computed tomography scan, magnetic resonance scan, intravascular ultrasound, etc. The Doppler ultrasonography is one of the initial investigations of choice for working up a suspected case of NCS [2]. Multidetector computed tomography (MDCT) scans can also efficiently detect NCS and can even distinguish between the NCS and NCP [11]. It appears that RHNP can also be diagnosed with advanced radiodiagnostic tools in the very initial subclinical stages [6]. As renal hilar vascular anatomy like RHNP could be the cause of renal venous hypertension resulting in pathological alterations in the kidney tissue, it should also be considered among the differential diagnosis of any case suggestive of renal venous hypertension due to probable extrinsic compression on RV. We presume that atypical varieties of NCP are underdiagnosed on account of nonavailability of necessary diagnostic facilities in most health care set ups. Existing studies highlighted that the incidence of all varieties of NCP and NCS together ranges to be around 10% [6]. Although RHNP causing atypical NCS is not reported yet, such possibility cannot be ruled out. Therefore, it is important to evaluate renal profile and pathological status of kidney if such variations are found in clinical settings.

## Conclusions

Renal hilar vascular anatomy exhibits variations and such a variant could be the cause of renal venous hypertension. Though nutcracker syndrome due to renal vein compression at the renal hilum is not well reported, a close monitoring of the renal hilar vascular pattern with appropriate imaging tests in suspected cases might yield useful information in understanding ‘renal hilar nutcracker phenomenon’ and its possible pathological consequences.

## Limitations

Clinical relevance of this finding should be confirmed in data obtained from well designed studies. Since this is an incidental observation noted during cadaveric dissection, actual incidence and clinical implications of these types of cases can only be explained clearly after observing in studies with appropriate sample size in clinical settings.
